# Antibiotic Resistance and Therapy for *H. pylori* Infection in Immigrant Patients Treated in Italy

**DOI:** 10.3390/jcm9051299

**Published:** 2020-05-01

**Authors:** Giulia Fiorini, Ilaria Maria Saracino, Angelo Zullo, Matteo Pavoni, Laura Saccomanno, Tiziana Lazzarotto, Rossana Cavallo, Guido Antonelli, Berardino Vaira

**Affiliations:** 1 Department of Medicine and Surgery Sciences, University of Bologna, 40138 Bologna, Italy; giulia.fiorini@fastwebnet.it (G.F.); saracinoilariamaria@gmail.com (I.M.S.); matteo.pavoni@studio.unibo.it (M.P.); laura.saccomanno@studio.unibo.it (L.S.); 2 Gastroenterology Unit, ‘Nuovo Regina Margherita’ Hospital, 00153 Rome, Italy; zullo66@gmail.com; 3 Department of Molecular Medicine, Sapienza University of Rome, 00185 Rome, Italy; guido.antonelli@uniroma1.it; 4 Department of Experimental, Diagnostic and Specialty Medicine, University of Bologna, 40138 Bologna, Italy; tiziana.lazzarotto@unibo.it; 5 Microbiology and Virology Unit, University Hospital Città Della Salute e Della Scienza di Torino, 10126 Turin, Italy; rossana.cavallo@unito.it

**Keywords:** *Helicobacter pylori*, immigrants, resistance, therapy

## Abstract

*Background*: *Helicobacter pylori* (*H. pylori*) infection is the leading cause of both peptic ulcers and gastric tumors, including low-grade MALT-lymphoma and adenocarcinoma. Although it is decreasing in developed countries, *H. pylori* prevalence remains high in developing areas, mainly due to low socio-economic levels, and the potential consumption of contaminated water. Moreover, a different pattern of primary antibiotic resistance is expected in their *H. pylori* isolates, potentially affecting the efficacy of standard eradication therapies. Indeed, a previous study showed the eradication rate following triple therapy was distinctly lower in dyspeptic *H. pylori* infected immigrants living in Italy as compared to Italian patients. *Aims*: to evaluate the resistance pattern in *H. pylori* isolates from immigrant patients in Italy, and the success rate of first-line therapy in these patients. *Materials and Methods*: This retrospective study evaluated data of consecutive immigrant patients, diagnosed with *H. pylori* infection in a single center (Bologna, Italy) between January 2009 and January 2019. Patients underwent first-line therapy with either sequential or Pylera^®^ (Allergan USA, Inc. Madison, NJ, USA) therapy. *Results*: A total of 609 immigrants were diagnosed with *H. pylori* infection during the study period, but 264 previously received an eradication therapy. Therefore, the study was focused on 294 out of 345 naïve patients with a successful bacterial culture with antibiogram. Latin America immigrants had the highest overall resistance rate. Levofloxacin resistance rate was significantly higher in Latin Americans and Asians as compared with Europeans. Based on resistance patterns, sequential therapy showed a clear decreasing trend in eradication rates. Conclusions: while antibiotic resistance rates are generally increasing worldwide, Pylera^®^ seems to achieve a good performance as first-line treatment in all naïve foreigner patients, except for Africans.

## 1. Introduction

*Helicobacter pylori* (*H. pylori*) infection is the leading cause of both peptic ulcers and gastric tumors, including low-grade MALT-lymphoma and adenocarcinoma [1]. Although it is decreasing in developed countries, *H. pylori* prevalence remains high in developing areas, mainly due to low socio-economic level, and the potential consumption of contaminated water [2]. Infected patients who move from their country retain the infection in the developed countries and usually present more severe clinical features, such as significant higher prevalence of peptic ulcers [3]. Moreover, a different pattern of primary antibiotic resistance is expected in their *H. pylori* isolates [4], potentially affecting the efficacy of standard eradication therapies. Indeed, a previous study showed the eradication rate following triple therapy was distinctly lower in dyspeptic *H. pylori* infected immigrants living in Italy as compared to Italian patients [5]. Moreover, a recent meta-analysis, showed that immigrants from regions with a high incidence of gastric cancer maintain a higher risk of gastric cancer and related mortality, therefore they can constitute high-risk populations that need specific prevention and therapeutic interventions [6]. This study aimed: (a) to evaluate the resistance pattern in *H. pylori* isolates from immigrant patients in Italy, and (b) the success rate of first-line therapy in these patients.

## 2. Materials and Methods

### 2.1. Patients

This retrospective study evaluated data of consecutive immigrant patients, diagnosed with *H. pylori* infection in a single center (S. Orsola Hospital, Bologna, Italy) between January 2009 and January 2019. The exclusion criteria were: (1) age <18 years; (2) previous gastric surgery; (3) use of proton pump inhibitor (PPI) or antibiotics in the 2 weeks before the endoscopy; (4) known allergy to antibiotics. All patients underwent upper endoscopy, five (two from the antrum, one from incisura angularis and two from the corpus) biopsy specimens were taken for histology, and one additional antral biopsy was used for bacterial culture and antibiotic susceptibility test. Based on endoscopic reports, for the purposes of the study, patients with either a peptic ulcer (ulceration ≥5 mm in diameter) or mucosal erosions (superficial lesion <5 mm) in the stomach or duodenum were grouped together as “peptic ulcer disease” (PUD). Non-ulcer dyspepsia was diagnosed when no macroscopic lesions were detected at endoscopy and patients were included in “non-ulcer disease” (NUD) group. All participants provided written informed consent before procedure.

### 2.2. Antibiotics Susceptibility Testing

Biopsy specimens collected for bacterial culture were streaked immediately onto commercial selective medium Pylori Agar (BioMérieux Italia S.p.A., Florence, Italy). The plates were incubated under microaerobic conditions at 37 °C for 3–5 days. Once incubated, the colonies resembling *H. pylori* were identified by oxidase, catalase, and urease tests. Suspensions from the primary plates were prepared in sterile saline solution to McFarland opacity standard 4, approximately 10^9^ colony forming units (CFU)/mL to perform an E-Test (BioMérieux Italia S.p.A.). A total of three agar plates for every *H. pylori* strain were streaked in three directions with a swab dipped into each bacterial suspension to produce a lawn of growth. Three E-Test strips (clarithromycin 0.016–256 ug/mL, metronidazole 0.016–256 ug/mL, and levofloxacin 0.008–32 ug/mL) were placed each onto a separate plate, which was incubated immediately in a microaerobic atmosphere at 37 °C for 72 h. A fourth plate was used as positive control. Clarithromycin, metronidazole, and levofloxacin resistance break points for the minimal inhibitory concentration (MIC) are greater than 0.5 mg/L, greater than 8 mg/L, and greater than 1 mg/L, respectively, according to the updated recommendations of the European Committee on Antimicrobial Susceptibility Testing (EUCAST) [4]. Since 2015 Eucast has established that clarithromycin MICs between 0.25 ug/mL and 0.5 ug/mL were to be considered as “indeterminate”, suggesting not to administer the drug in this case, so these strains were considered as “resistant” in this study [6].

### 2.3. Eradication Therapies

According to Italian and European Guidelines [7,8,9] patients underwent first-line therapy with either sequential or Pylera^®^ (Allergan USA, Inc. Madison, NJ, USA) therapy. Patients were enrolled consecutively, therefore the date of recruitment was crucial to be allocated to one of treatments. In detail, before 2016 sequential therapy was used. Following 2016, Pylera^®^ therapy was mainly used (Pylera^®^ has only been available in Italy since 2016). Sequential therapy included 5 days of a dual therapy with 40 mg esomeprazole twice a day (before breakfast and dinner) and 1000 mg of amoxicillin twice a day (after breakfast and dinner) followed by 5 days of a triple therapy with 40 mg esomeprazole twice a day (before breakfast and dinner) and clarithromycin 500 mg and metronidazole 500 mg both twice a day (after breakfast and dinner). From 2016 when Pylera^®^ was available in Italy, a three-in-one capsule containing 140 mg bismuth subcitrate potassium, 125 mg metronidazole, and 125 mg tetracycline. Therefore, Pylera^®^ therapy was a 10 days quadruple therapy with 20 mg omeprazole twice a day (before breakfast and dinner) plus three Pylera^®^ capsules four times a day (after breakfast, lunch, dinner and before bedtime). Treatment success was evaluated by using a standard ^13^C-Urea Breath Test (UBT) performed 6 to 8 weeks after treatment ended. In the event of an early interruption of eradication therapy, UBT was performed when at least 7 days of treatment were token. Patients undergoing therapy for fewer than 7 days or who did not undergo UBT testing after treatment were considered as drop-outs.

### 2.4. Statistical Analysis

Means and their 95% confidence intervals were calculated. Eradication rates were calculated both by intention-to-treat (ITT) analysis, including all treated patients, and by per-protocol (PP) analysis, including patients who took more than 90% of their medications and completed follow-up evaluation. Comparisons among patient subgroups were performed using the Fisher exact Chi-square test, as appropriate, and the Odd Ratios were calculated. A P level less than 0.05 was considered statistically significant. Statistical analysis was performed with MedCalc19.1 (MedCalc Software Ltd, Ostend, Belgium).

## 3. Results

A total of 609 immigrants were diagnosed with *H. pylori* infection during the study period, but 264 previously received an eradication therapy. Therefore, the study was focused on 294 out of 345 naive patients with a successful bacterial culture and antibiogram. Clinical and demographic characteristics were provided in Table 1. The immigrants were coming from East Europe (N = 165), Asia (N = 44), Africa (N = 54) and Latin America (N = 31).

The patterns of primary resistance in *H. pylori* isolates are reported in Table 2, and the distribution among different countries in Table 3.

As shown, Latin America immigrants had the highest overall resistance rate. Moreover, levofloxacin resistance rate was significantly higher in Latin Americans (*p* = 0.028; OR: 2.42; 95% CI = 1.1–5.3) and Asians (*p* = 0.015; OR 2.44, 95% CI = 1.22 to 4.86) as compared with Europeans.

Regarding therapy, the eradication rates achieved by sequential therapy were 84% PP (95% CI 78.2–88.7) and 70.6 at ITT (95% CI 64.5–76.2) analyses. Eradication rates for Pylera^®^ were 95.7% at PP (95% CI = 85.5–99.5) and 91.8% at ITT (95% CI = 80.4–97.7) analyses.

The success rate with Pylera^®^ was significantly higher than that of sequential therapy at both PP (*p* = 0.035; OR: 4.3, 95% CI = 1.01–18.56) and ITT (*p* = 0.001; OR 4.68, 95% CI = 1.62–13.49) analyses. Based on resistance patterns, sequential therapy showed a clear decreasing trend in eradication rates, from 87.4% at PP (80.6% at ITT) in drug susceptible strains to 74.4% at PP (54.7% at ITT) in strains resistant towards both clarithromycin and metronidazole. Bacterial eradication rates following Pylera^®^ remained stable (Table 4).

As shown in Table 5, the analysis performed based on the Continent of birth showed that South-Americans tended to have the lowest eradication rate with sequential therapy, while Africans showed the lowest eradication rate with Pylera^®^.

## 4. Discussion

*H. pylori* is the most common chronic bacterial infection in the world, and it is a well-known risk factor for gastric cancer. Infection prevalence ranges from 20% to 35% in most high-income countries to 60% to 90% in developing countries [9]. As previously demonstrated, region of origin is an important determinant of gastric cancer risk as individuals may import their genetic and *H. pylori* strain-associated risk factors that are further modulated by diet, microbiome, behavioural and environmental factors [10,11,12]. Immigrants from developing geographic areas have a high prevalence of *H. pylori* infection as compared to the local population [13]. Therefore, screening and treatment of *H. pylori* in these high-risk populations has been suggested to reduce the burden of gastric cancer and peptic ulcer disease and have also been shown to be cost effective [14].

Standard triple therapy for *H. pylori* in many countries is now less than 80% effective, primarily due to clarithromycin resistance. Therefore, other options can be more effective including sequential therapy and longer courses (10–14 days) of quadruple therapy, including bismuth, tetracycline, metronidazole and a PPI [15,16].

This study aimed to assess the resistance pattern and eradication performance of first-line therapies for *H. pylori* eradication in immigrants. Our results showed that South-Americans had the highest overall resistance rate, and that levofloxacin resistance rate was significantly higher in South-Americans and Asians as compared with Europeans. This observation could depend on the use of antibiotics in the general population of different geographic areas. South-Americans had also the lowest eradication rates for sequential therapy, instead Africans showed the lowest eradication rates for Pylera^®^. Although it is difficult to explain these findings, it is known that several factors are involved in the *H. pylori* therapy failure. Indeed, besides antibiotic resistance pattern, compliance to therapy, smoking habit, gastroduodenal disease, bacterial strain sub-type, and cytochrome P450 polymorphisms may be involved [17].

Focusing on eradication rates, sequential therapy showed a clear decreasing trend in strains resistant to both clarithromycin and metronidazole while Pylera^®^ eradication rates remained stable. The difference in the eradication rate between the two therapy regimens could depend on the different composition. Indeed, Pylera^®^ has a higher daily metronidazole dose than the sequential therapy and, consequently, it could be more capable of overcoming metronidazole resistance, highly prevalent in *H. pylori* isolates in developing areas. Moreover, the use of tetracycline might be more suitable than clarithromycin in immigrants with a high proportion of strains with clarithromycin resistance. [18]. Data on eradication rates using first-line treatments in naïve Italian patients diagnosed in the same period were published in an ad hoc study [19]

This study may have some potential limitations. Data on previous exposure to antibiotics were not available and, if available, they are subjected to significant recall bias. Our findings were also based on patients enrolled in only single hospital, therefore the study population may not adequately mirror the general population. Furthermore, data on compliance were not recorded and, even if the enrolment was consecutive, possibility of selection bias cannot theoretically be excluded. On the other hand, the literature on this topic is still scanty and few evidences regarding the best therapeutic approach as first-line in foreigners.

## 5. Conclusions

While antibiotics resistance rates are generally increasing worldwide, Pylera^®^ seems to achieve a good performance as first-line treatment in all naïve foreigner patients, except for Africans.

## Figures and Tables

**Table 1:** Demographic and clinical characteristics of patients.

Population Features	Number	%	95% CI
Patients	294		
Male	99	33.7	25.5–39.2
Female	195	66.3	60.7–71.5
Age range, mean	18–82, 41.3		
BMI range, mean	15.5–42.6, 25.1		
Active smokers (yes)	62	21.1	16.8–26.1
Alcohol consumption (yes)	31	10.5	7.5–14.5
Cardioaspirin (yes)	10	3.4	1.8–6.1
Familiarity for gastric cancer (yes)	25	8.5	5.8–12.2
NUD	256	87.1	82.7–90.4
PUD	36	12.2	8.9–16.4
MALT-Lymphoma	2	0.7	0.2–2.4

95% CI: 95% Confidence Interval. BMI: Body Mass Index. NUD: Non-Ulcer Disease. PUD: Peptic Ulcer Disease. MALT: mucosal-associated lymphoid tissue.

**Table 2:** Pattern of primary resistance in *H. pylori* isolates.

N = 294	N	%	95% CI
ClaR, MzR, LevoR	34	11.6	8.1–15.8
ClaR, MzR, LevoS	29	9.9	6.7–13.9
ClaR, MzS, LevoR	12	4.1	2.1–7.0
ClaR, MzS, LevoS	28	9.5	6.4–13.5
ClaS, MzR, LevoR	28	9.5	6.4–13.5
ClaS, MzR, LevoS	39	13.3	9.6–17.7
ClaS, MzS, LevoR	18	6.1	3.7–9.6
ClaS, MzS, LevoS	106	36.1	30.6–41.8
Cla R tot	103	35.0	29.6–40.8
Mz R tot	130	44.2	38.5–50.1
Levo R tot	92	31.3	26.0–36.9
RR tot	63	21.4	16.9–26.6

Cla: clarithromycin. Mz: metronidazole. Levo: levofloxacin. S: susceptible. R: resistant. RR: double clarithromycin/metronidazole resistance. 95%CI: 95% Confidence Interval.

**Table 3:** Prevalence of antibiotic resistance according to Continent.

	Europe (165)	%	Africa (54)	%	South-America (31)	%	Asia (44)	%
**ClaR, MzR, LevoR**	14	8.5	8	14.8	6	19.3	6	13.6
**ClaR, MzR, LevoS**	22	13.3	3	5.5	2	6.5	2	4,5
**ClaR, MzS, LevoR**	5	3.0	1	1.8	3	9.7	3	6.8
**ClaR, MzS, LevoS**	14	8.5	8	14.8	3	9.7	3	6.8
**ClaS, MzR, LevoR**	13	7.9	5	9.2	2	6.5	8	18.2
**ClaS, MzR, LevoS**	23	14.0	8	14.8	3	9.7	5	11.3
**ClaS, MzS, LevoR**	10	6.1	2	3.7	3	9.7	3	6.8
**ClaS, MzS, LevoS**	64	38.8	19	35.2	9	29.0	14	31.8
**Cla R tot**	55	33.3	20	37.0	14	45.1	14	31.8
**Mz R tot**	72	43.6	24	44.4	13	41.9	21	47.7
**Levo R tot**	42	25.4	16	29.6	14*	45.1	20*	45.4
**RR tot**	36	21.8	11	20.3	8	25.8	8	18.2

Cla: clarithromycin. Mz: metronidazole. Levo: levofloxacin. S: susceptible, R: resistant. * *p* < 0.05 compared with European patients.

**Table 4:** Eradication rates according to the bacterial resistance pattern.

Resistance Status	Therapy	N	PP	95% CI	ITT	95% CI
ClaS, MzS (124)	Sequential	103	87.4	79.2–92.6	80.6	71.9–87.1
	Pylera®	21	94.7	75.4–99.0	85.7	65.3–99.0
ClaR, MzR (63)	Sequential	53	74.4	58.9–85.4	54.7	41.4–67.3
	Pylera®	10	90.0	59.6–98.2	90.0	59.6–98.2
ClaR, MzS (40)	Sequential	33	89.7	73.6–96.4	78.8	62.2–89.3
	Pylera®	7	100.0	64.5–100.0	100.0	64.5–100.0
ClaS, MzR (67)	Sequential	56	81.4	67.4–90.2	62.5	49.4–73.9
	Pylera®	11	100.0	74.1–100.0	100.0	74.1–100

Cla: clarithromycin. Mz: metronidazole. S: susceptible, R: resistant. PP: per protocol analysis. ITT: intention to treat analysis. CI: Confidence Interval.

**Table 5:** Eradication rates according to the continent of birth.

Continent of Birth	Therapy	PP	95%CI	ITT	95%CI
Europe	Sequential	84.5	76.8–89.9	72.0	63.9–78.9
	Pylera®	96.3	81.7–99.3	89.6	73.6–96.4
Africa	Sequential	83.3	68.1–92.1	65.2	50.7–77.3
	Pylera®	87.5	59.9–97.8	87.5	59.9–97.8
America	Sequential	80.0	58.4–91.9	69.6	49.1–84.4
	Pylera®	100.0	67.6–100.0	100.0	67.6–100.0
Asia	Sequential	85.3	69.8–93.5	72.5	57.2–83.9
	Pylera®	100.0	51.1–100.0	100.0	51.1–100.0

PP: Per Protocol analysis. ITT: intention to treat analysis. CI: Confidence Interval.
